# Effect of diet on the structure of animal personality

**DOI:** 10.1186/1742-9994-12-S1-S5

**Published:** 2015-08-24

**Authors:** Chang S Han, Niels J Dingemanse

**Affiliations:** 1 Behavioural Ecology, Department of Biology, Ludwig-Maximilians University of Munich, Planegg-Martinsried, Germany; 2 Research Group Evolutionary Ecology of Variation, Max Planck Institute for Ornithology, Seewiesen, Germany

**Keywords:** behavioural syndrome, gene-environment interaction, genetic correlation, intake target, nutrition, personality, quantitative genetics

## Abstract

There is increasing interest in the proximate factors that underpin individual variation in suites of correlated behaviours. In this paper, we propose that dietary macronutrient composition, an underexplored environmental factor, might play a key role. Variation in macronutrient composition can lead to among-individual differentiation in single behaviours (‘personality’ ) as well as among-individual covariation between behaviours (‘behavioural syndromes’ ). Here, we argue that the nutritional balance during any life stage might affect the development of syndrome structure and the expression of genes with pleiotropic effects that influence development of multiple behaviours, hence genetic syndrome structure. We further suggest that males and females should typically differ in diet-dependent genetic syndrome structure despite a shared genetic basis. We detail how such diet-dependent multivariate gene-environment interactions can have major repercussions for the evolution of behavioural syndromes.

## Introduction

**Figure 1:**
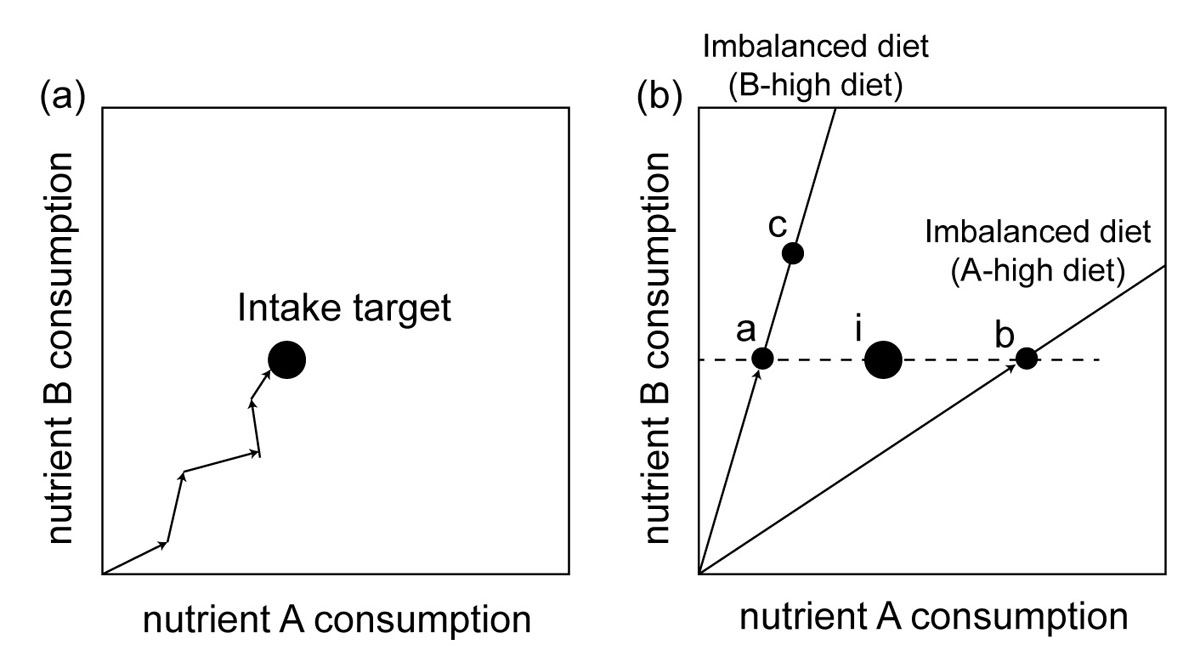
**The geometry of nutritional decisions.** (a) Animals can reach a nutritional intake target (ratio of nutrient A consumption relative to B consumption) by switching nutritionally complementary foods. (b) However, the intake target (point i) cannot be reached when animals are forced to forage on imbalanced food sources. When animals must satisfy the requirement of nutrient B, they suffer a deficit of nutrient A (point a) or an excess of nutrient A (point b). Otherwise, animals suffer both an excess of nutrient B and a deficit of nutrient A (point c). The illustration is modified from figure 1 in Ref. [1].

Animals require multiple nutrients for the process of somatic maintenance, growth, development and reproduction [1, 2]. Typically, individuals do not aim to consume all foods maximally. Instead, they usually balance the intake of key nutrients. Animals have multiple behavioural and physiological regulatory mechanisms to absorb the optimal mixture of nutrients to meet energetic and structural needs, which is referred to as their ***intake target***[3, 4] (Figure 1a). An optimal mix of nutrients has been hypothesised to facilitate optimal growth (cf. fitness) [2].

When it is inevitable to consume nutritionally imbalanced diets, animals can solve the problem by selectively consuming multiple types of foods (i.e. nutritionally complementary foods; [5, 6]). However, when faced with a single nutritionally imbalanced diet, animals might not reach their intake target (Figure 1b). Under such conditions, animals typically consume as much as needed to acquire sufficient amounts of the most important nutrient (i.e., in terms of fitness returns), and thus under- or over-consume certain nutrients (Figure 1b, [7]). Although excess nutrients can be utilised and selectively excreted, individuals may not be able to void the excesses if the degree of imbalance exceeds the capacity to excrete them. For example, when consuming carbohydrate-biased food, animals ingest surpluses of carbohydrates to acquire adequate amounts of protein. Surplus amounts of carbohydrates are, in turn, typically converted to lipids and stored in the body [8]. Both deficits and surpluses of nutrients can strongly influence behaviour, physiology, reproductive output, growth, or survival [9–17].

In ecology and evolution, there has been considerable interest in the effect of nutritional condition on life-history traits, morphology and longevity [11, 13, 17–21]. Nutrition can also be regarded as a major component of behavioural development since it represents a key environmental factor (see [22]). Nutritional conditions early in an individual's life can provide reliable cues on the optimal level of various behaviours expressed later in life [11, 23–27]. Individuals reared on diets of high quality are, for example, generally more active than individuals feeding on a low-quality diet [22]. Malnutrition can also affect the level of boldness and aggression [28].

Despite the well-known existence of nutritional effects on behavioural development, only a few studies have used experiments to investigate effects of macronutrient composition (and their balance) on behaviour and its development (i.e., by controlling the amount of certain nutrients or the ratio of them), with most research to date focusing on invertebrates (insects and spiders: Table 1, [9–11, 13, 14, 16, 29]). The expression of reproductive behaviour (i.e., calling effort) of male crickets (*Teleogryllus commodus*) is, for example, increased by a low-protein high-carbohydrate diet [13] because carbohydrates represent the main energy source for metabolic processes, allowing muscle and tissue to exert high metabolic activity. *Drosophila* males, furthermore, increase their mating frequency and courtship when fed on a protein-high diet [9, 10]. The incidence of cannibalism in Mormon crickets (*Anabrus simplex*) decreases when individuals can access high-protein food sources [29]. Given the diversity of nutritional requirements that different kinds of animal species (i.e., herbivores, carnivores and omnivores) face, intake targets, and their effects on behavioural phenotypes, can vary greatly across species.

**Table 1:** Effect of macronutrient diet composition on the expression of behaviour in arthropods.

Organism	Macronutrient	Effect on behaviour ^a^	References
**cricket**	carbohydrate	male calling effort (+)	[11, 13, 16]
**wolf spider**	protein (+ vitamins)	female aggression (+)	[14]
***Drosphila***	protein	male courtship (+)	[10]
**Fruit fly**	protein	male mating frequency (+)	[9]
**Mormon cricket**	protein	cannibalism (-)	[29]

^a^. Effect of high nutrient intake on behavioural expression: ‘(+)’ indicates that the expression of the behaviour is increased when the macronutrient intake is high.

† This Table excludes research on effects of diet quantity (e.g. calories, food-abundance/deficiency) and research on effects of diet quality in which macronutrients are not completely controlled for (e.g. different types of prey or prey at different ages).

Nutritional balance can also affect the extent of individual differentiation in behaviour (i.e., among-individual variance), for example, because diet can induce long-term effects in morphology or physiology, hence behaviour. For example, deficiencies or excesses of certain nutrients might facilitate or attenuate the expression of behaviour of most individuals, thereby increasing or decreasing the among-individual variance in behaviour. Although one study showed that the extent of among-individual differentiation in behaviours was not a function of nutritional environment [15], the relationship between diet and among-individual variation in behaviour remains largely unexplored.

Nutritional balance can also affect the amount of among-individual covariance between functionally distinct behaviours. Behavioural correlations exist not just due to pleiotropic effect of genes (or linkage disequilibrium) but also due to pleiotropic environmental effects (such as macronutrient composition) [30]. The covariance between behaviours is thus shaped by the combined effects of environmental and genetic correlations (see below). Interestingly, environmental conditions can also affect the expression of gene pleiotropy, and thereby the strength of genetic correlations between behaviours (e.g. [31]). In certain nutritional environments, there might thus be more or less genetic variation expressed, leading to environment-specific heritability (cf. G×E) [32].

Furthermore, because of their shared genetic basis, males and females within a species are typically not thought to differ in the expression of genetic (co)variation in behaviours. However, given sexual differences in the optimal diet for fitness maximisation [33], we expect diet-dependent among-individual variation and covariation in behaviour to be sex-specific. We discuss how such sex-specificity should arise in our general discussion.

In this opinion paper, we discuss the effects of extreme biological scenarios where the composition of macronutrients in the nutritional environment is so biased that individuals are unable to consume the optimal amount of nutrients for the intake target (e.g. a protein-deficient environment). In the natural environment, this situation may occur when animals consistently stay within one habitat, which might occur when animals face restrictions in dispersion [22, 34, 35]. In such situations, individuals may be unable to avoid fitness costs associated with nutrient deficiencies or consumption of excesses of biased nutrients [1, 36]. Our aim is thus to explore how the level of nutritional imbalance might influence the genetic and environmental underpinning of suites of correlated behaviours. We propose that the composition of diet with respect to macronutrients can have major pleiotropic effects on suites of behaviours, thereby explaining why they might covary. We further introduce a quantitative genetics approach to empirically test how nutritional composition might affect (the interacting effects of) developmental and genetic factors underpinning behavioural syndromes.

## The structure of behavioural syndromes and environmental effects

Over the last decade, a large number of behavioural studies have shown that individuals of the same population differ consistently in their behaviour [37]. Individual differences in behaviour that are repeatable over time and across different contexts or situations are commonly referred to as ‘animal personality’ in the behavioural ecology literature [38]. The repeatable components of animal behaviour are often also correlated with each other across traits (meta-analysis: [39]), and such among-individual correlations are called ‘behavioural syndromes’ [40–44]. The most widely-documented example is the aggressiveness-boldness syndrome: individuals that are on average relatively aggressive towards conspecifics are also relatively active and bold towards predators, compared to less aggressive individuals. Behavioural syndromes may also include other functionally distinct behaviours, such as dispersal tendency, exploration, docility, cooperation, sociability, and mating strategy [45, 46].

**Figure 2:**
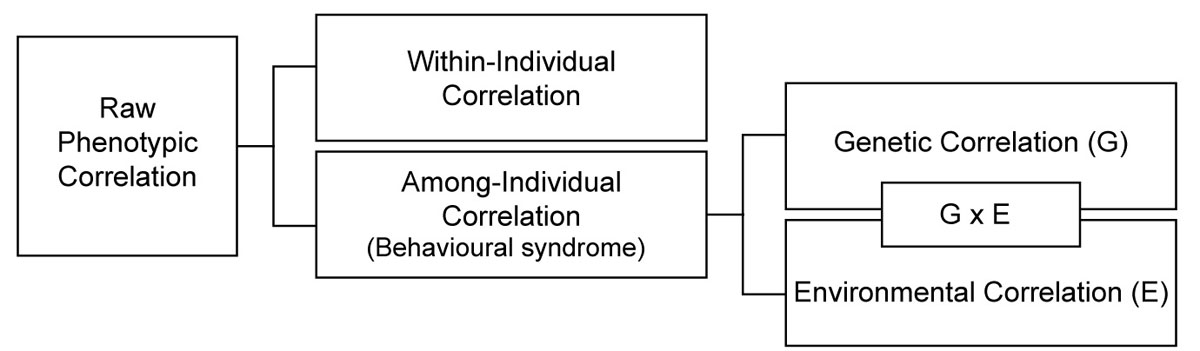
**The contribution of genetic and environmental factors in shaping behavioural syndromes.** A hierarchical diagram illustrating how raw behavioural correlations can be decomposed into within-individual and among-individual correlations. The among-individual correlation (behavioural syndrome) is the correlation between each individual's average phenotype across multiple behaviours. The within-individual correlation, in contrast, is the correlation between changes in multiple behaviours expressed within the same individual. Among-individual correlations are themselves affected by genetic effects (via pleiotropy or linkage disequilibrium) and environmental effects [40].

Behavioural correlations are the product of the joint influences of ‘among-individual’ and ‘within-individual’ correlations ([40, 44, 47], Figure 2). The among-individual correlation is the correlation between each individual's average phenotype across multiple behaviours, i.e. the correlation between the repeatable parts of behavioural traits. Within-individual correlations, in contrast, exist when within-individual plasticity is correlated across traits due to ‘integration of plasticity’ unless caused by correlated measurement errors. Behavioural syndromes refer to among-individual correlations rather than simple un-partitioned phenotypic correlations, and are estimated with considerable bias if within-individual correlations are not accounted for [40].

Among-individual correlations are themselves shaped by two main contributors: genetic (G) and environmental effects (E) [40] (Figure 2). First, genetic correlations between behavioural traits result in among-individual correlations because of gene pleiotropy (i.e., a single gene governs the expression of multiple behavioural traits) or linkage disequilibrium (i.e., genes affecting one behaviour are correlated with genes affecting another) [48]. Second, non-genetic (i.e., environmental) factors can also cause among-individual correlations, either when environmental factors have long-term effects on the development of multiple behavioural traits (e.g. due to environmental factors with pleiotropic effects) [49–53] or when environmental factors with short-term effects are themselves repeatable across individuals (also called ‘permanent’ environmental effects). The known occurrence of such environmental effects in natural populations [44, 54–56] implies that environmental conditions (such as feeding conditions in early life) can profoundly influence behavioural syndrome structure.

There is already considerable evidence for a major role of genetic correlations in shaping behavioural syndromes [41, 57, 58]. The contribution of permanent environmental correlations has, in contrast, largely been ignored (e.g. [40]). Permanent environment correlations may shape among-individual correlations independently from genetic correlations (leading to additive effects: G+E). Among-individual correlations can, by contrast, also result from interactions between genetic and long-lasting environmental effects (i.e. G×E). For example, environmental conditions can alter the expression level of a gene involved in a signalling pathway connected to a behaviour [59, 60], and affect the expression, hence heritability, of genetic variation in behaviour [56]. The strength of genetic correlations between behaviours can also depend on the environment when environmental conditions affect the gene expression related to the breakdown of a neurotransmitter, such as histamine, connected to multiple behaviours (e.g. aggression, exploration and boldness) [60, 61]. That is, if genes with pleiotropic effects are disproportionally more expressed in specific environments, genetic correlations become a function of the environment. Environmental factors, such as nutritional balance, might thus greatly affect the expression of genetic correlations that underpin behavioural syndromes [41].

## Multivariate effects of nutritional condition on behavioural phenotypes

Given that nutrition is known to affect behaviour and its development [9–11, 13–16], we predict that the composition of macronutrients might similarly represent an important environmental effect causing correlations between suites of behaviours (i.e., behavioural syndrome structure, Figure 2). Behavioural effects of excesses (or deficits) in consumption of single nutrients (relative to intake target, Figure 1b) likely depends on the functional context in which behaviour is expressed. For example, activity, exploration, dispersal and parental care are predicted to be sensitive to excesses and deficits of carbohydrate in the diet. This is because carbohydrates act as a main energy source used to ‘fuel’ the expression of such energetically demanding behaviours; high carbohydrate intake might also increase metabolic rate, thereby again facilitating the expression of energetically demanding behaviours [62]. Males exposed to high-carbohydrate diets might therefore be more active and explorative, and more willing to engage in active mate choice and courtship compared to individuals on low-carbohydrate diets. In addition, since sexual behaviours such as courtship or female resistance generally show condition dependence [63–66], such expression might also depend on nutrition. Similarly, sociality or cooperative behaviour is also predicted to be sensitive to the amount of carbohydrate or protein in the diet. Since cooperative/social behaviours are regulated by neuroendocrine mechanisms [67], the known adverse effect of poor nutrition on neuromuscular development [68–72] could influence the expression of cooperative/social behaviours. Aggressive behaviour is particularly predicted to vary with level of protein intake [14]. Protein-deprived individuals are expected to be bolder and more aggressive than those under balanced diets because they have less to lose in terms of future fitness expectations (following ref. [73]). In cannibalistic species such as Mormon crickets, protein deficiency is also likely to increase the expression of aggressive behaviour because the deficit of proteins leads to increased frequency of cannibalism [29].

**Figure 3:**
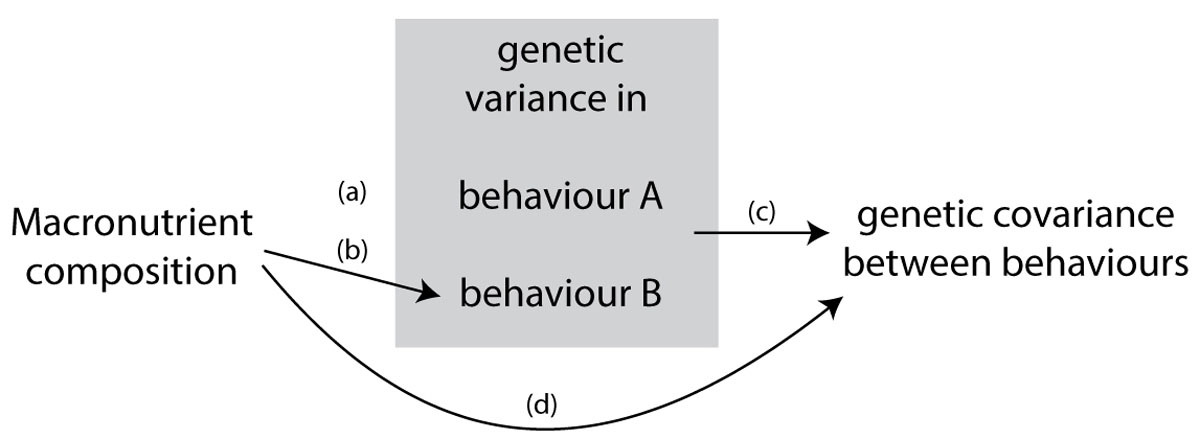
**A graphical prediction of effects of macronutrient composition on the expression of genetic variance and covariance in multiple behaviours**. Macronutrient composition is predicted to affect the expression of genetic variance in certain behaviours (arrows a and b), and correlation between behaviours (arrow c). Effects of macronutrient composition likely depend on the type of context in which behaviour is expressed (arrows a and b). Moreover, genetic covariation between behaviours could also be directly determined by macronutrient composition via pleiotropic gene actions without changing among-individual variance (arrow d).

The extent to which intake of macronutrients is balanced is also expected to affect the expressed amount of among-individual variance in behaviour (Figure 2, 3). That is, in one diet treatment the repeatability might be higher or lower compared to another. For example, in rich environmental conditions with *ad libitum* balanced food intake, among-individual variation in behavioural traits might be much higher than in a nutritionally impoverished environment (c.f. with imbalanced food availability) because the expression of genetic variation is typically increased under favourable conditions ([74, 75], but see [76]). This is because under nutritionally imbalanced conditions, most individuals fail to get enough resources (e.g. carbohydrate or protein) to express costly behaviours, resulting in decreased individuality in behaviour. Otherwise, the imbalanced composition of macronutrients could also make all individuals increase the expression of types of behaviour that enable them to escape nutritional deficiency (e.g. foraging behaviour), which would result in increased among-individual variance in behaviour. Therefore, given the effect of the composition of macronutrients on the expression of among-individual variation in behaviour, we expect that the dietary balance plays a key role in shaping among-individual correlations between behaviours (i.e. environment-specific behavioural syndromes).

## Diet-dependent genetic correlations underlying behavioural syndromes

Quantitative genetic analyses can be utilized to simultaneously quantify the relative importance of environmental effects (e.g. due to diet) versus diet-specific genetic correlations underlying among-individual behavioural correlations. Quantitative genetic analyses and animal models enable estimation of the **G**-matrix (a tabulation of additive genetic variances and additive genetic covariances) which has an important role in constraining evolution of phenotypes in response to selection or genetic drift [77]. Despite its central role, the stability of the **G**-matrix remains incompletely understood [78–82]. Although **G**-matrices are highly conserved among populations of certain species [78], empirical studies conducted in natural and laboratory populations show that **G** can change [83–89]. It is also largely unknown whether **G**-matrices for behavioural traits vary across environments. Given that behavioural traits are likely to be subject to permanent environmental effects, we predict that **G**-matrices for behavioural traits can change across different environments such as those differing in availability of different types of nutrients (i.e., G×E).

**G**-matrices measured for different macronutrient composition environments can be used to study cross-environment (dietary balances) genetic correlations as well as diet-specific genetic correlations, and reveal the genetic architecture and stability of the **G**-matrix; this is a powerful approach that has rarely been applied in the context of behaviour (but see [32]). The genetic correlation between morphological or life history traits (e.g. development time, body size, weight, longevity, fecundity etc.), for instance, can differ considerably between nutritional environments (e.g. calories) (see reviews, [90, 91]). Likewise, as the amount of among-individual variance in behaviour is expected to vary between environments, the amount of genetic variance within a given behaviour and covariance between behaviours (i.e. genes with pleiotropic effects), are also expected to depend on the environment [30, 92] (Figure 3). For example, as the phenotypic and genotypic variance in traits increase under favourable conditions [74], nutritional imbalance can decrease the amount of genetic variation in behavioural expression. On the other hand, the amount of genetic variation in behaviour expressed in nutritionally imbalanced environments could far exceed that expressed in nutritionally balanced environments when imbalanced nutritional compositions increase the expression of behaviour (e.g. increased foraging to escape nutritional deficiency or increased cannibalism). Furthermore, nutritional imbalance can similarly assert a direct influence on the covariation between behaviours [91, 93]. This implies that the expression of genes with pleiotropic effects can differ in direction between nutritionally imbalanced and balanced environments.

Imagine, for example, an insect species where protein-deficient diet increases aggression towards conspecifics but decreases reproductive behaviours ([9, 10, 14, 29], Table 1). In such a species, protein intake can affect the expression of genetic variation in behaviour and genetic correlations among behaviours. Proteins are a source of nitrogen for growth and maintenance of tissues, production of enzymes or spermatophores, as well as a source of metabolic energy via gluconeogenesis. Thus individuals faced with protein-deficient diet could express a high incidence of cannibalism [29] and fail to produce gametes of high quality [17, 94, 95], which could also induce less active reproductive behaviours (e.g. less courting) [9, 10]. As a result, if fed protein-deficit diets, males are likely to differ in their strategy to express aggressive behaviours using limited proteins, thereby leading to more genetic variance in aggressive behaviours. In contrast, genetic variation in reproductive behaviours might decrease because the limitation of overall protein availability in the food decreases reproductive activity. In addition to its effect on the expression of genetic variation, the level of protein intake can also directly influence level of genetic covariation among behaviours. This would occur if diet affects particular genes with pleiotropic effects on the expressions of multiple behaviours only. Therefore genetic variation in behaviours sensitive to protein intake (e.g. courtship behaviour, aggression) is likely to vary as a function of the amount of protein in the diet. The composition of diets with respect to macronutrients could also affect the direction of genetic correlations among behaviours via pleiotropic gene actions. Hence, genetic correlations between types of behaviours are predicted to be diet-specific.

In summary, genetic correlations can thus differ between balanced-nutrient and imbalanced-nutrient conditions because of gene-environment interactions acting on behavioural correlations. This implies that the genetic correlation should be diet-specific, which has consequences for the evolutionary potential of behavioural traits [41]. Such environment-specific genetic correlations would contribute to the (in)stability of behavioural **G**-matrices across dietary balances and also drive within-species polymorphism in behaviours when populations are faced with a diversity of nutritional habitats.

## Sex differences in diet-dependent genetic correlations

Since males and females share a common genetic basis, sex differences in environment-dependent genetic correlations might generally be rare. However, sex-specific alleles and genetic variance in traits can occur when strong selection on shared traits in one sex displaces the other sex from its phenotypic optimum in spite of genetic constraints [96]. In turn, such sex-specificity in expression contributes to the evolution of sexual dimorphism [97–99]. In the same way, sex-specific environment-dependent genetic correlations can also arise as a form of sexual dimorphism [89, 99–103].

Males and females differ in their optimal diet because the balance of nutrients for the optimal performance and fitness maximisation is normally sex-specific [33]. In crickets, for example, females prefer protein-rich diets for egg production, whereas males prefer carbohydrate-rich diet for energetically demanding courtship behaviour. Thus male crickets raised on carbohydrate-biased diet accumulate body lipid more readily than females [13]. Another example comes from caterpillars, where males prefer diets with a balanced protein/carbohydrate ratio while female caterpillars prefer a more protein-biased diet [104, 105]. In addition, female caterpillars utilise the excess of proteins more efficiently than males [104]. This indicates that intake targets and physiological systems that animals use to deal with the nutritional imbalance might typically differ between sexes.

Given a striking sex difference in responses to environmental stress from nutritional imbalance, we might expect relatively weak cross-sex genetic correlations between behaviours. In a protein-deficient environment, females are unable to produce many eggs and suffer fitness costs [13]. In contrast, since small amounts of protein suffice males to produce sperm, males do not suffer equally from protein-deficiency. Thus, based on the fact that protein is an important resource for females to produce eggs, protein-deficient environments will be more stressful for females. Thus cross-diet environment genetic correlations of females may be ephemeral because the correlation would easily break down under a protein-deficient environment. Cross-diet environment genetic correlations of males, however, may instead be fixed.

Therefore, behaviours involved in reproduction are likely to show sex differences in diet-dependent genetic correlations. In particular, dietary effects on sex-specific variation in one behavioural trait can result in correlated effects on sex-specific covariation between behavioural traits via pleiotropy. Because of sex-specific pleiotropy, we thus expect that males and females differ in diet-dependent among-individual variation in behaviours and covariation among them. Furthermore, given that the effects of diet may strongly depend on the specific life-history trajectories (or strategies) of each species, the magnitude of sex differences in diet-dependent genetic correlations should be species-specific.

## Conclusions

Animals need multiple nutrients to maximize their fitness. A variety of nutrients must be ingested in an optimal blend required for best performance. If the animal fails to attain the optimal balance of nutrients, nutritional imbalance will likely exert an effect on the expression of behavioural correlations, morphology (e.g. body composition), and physiology at the among-individual level. Though behavioural traits are heritable [106–108], there is a basic lack of understanding of how environmental effects interact with genetic variation to influence the development of behaviour [56]. Our conceptual framework addressing effects of dietary composition with respect to macro nutrients on the development of behavioural syndromes highlights how the expression of behavioural phenotypes can be altered by environmental stimuli, and reveals the role of nutritional balance on the plastic expression of behavioural genetic correlations. Research on nutrition and behavioural syndromes will thus spur a new wave of research beyond simply documenting behavioural correlations and testing the mechanisms that shape variation in behavioural syndromes and its underlying genetics. Studies on sex-specific genetic covariance, finally, represent an important step toward understanding intra-locus sexual conflict as well as the evolutionary basis of individual differences in behaviour.

## Declarations

Publication costs for this article were funded by the German Research Foundation (FOR 1232) and the Open Access Publication Fund of Bielefeld University and Muenster University.
